# Growth and Survival of Acid-Resistant and Non-Acid-Resistant Shiga-Toxin-Producing *Escherichia coli* Strains during the Manufacture and Ripening of Camembert Cheese

**DOI:** 10.1155/2009/653481

**Published:** 2009-02-11

**Authors:** M. P. Montet, E. Jamet, S. Ganet, M. Dizin, S. Miszczycha, L. Dunière, D. Thevenot, C. Vernozy-Rozand

**Affiliations:** ^1^Unité de Microbiologie Alimentaire et Prévisionnelle (UMAP), Département de Santé Publique Vétérinaire, Ecole Nationale Vétérinaire de Lyon, Université de Lyon, 69280 Marcy l'étoile, France; ^2^Pôle Microbiologie d’Intérêt Laitier (MIL), L'institut Technique du Lait et des Produits Laitiers (Actilait), Route des champs laitiers 419, 74801 La Roche-sur-Foron, France

## Abstract

Growth and survival of acid-resistant (AR) and non-acid-resistant (NAR) Shiga-toxin-producing *Escherichia coli* (STEC) strains were investigated during the manufacture and ripening of microfiltered milk Camembert cheeses. The induction of acid resistance of the STEC strains in cheeses was also studied. Six different mixtures of AR and/or NAR STEC strains were inoculated separately into microfiltered milk at a level of 10^3^ CFU mL^−1^. The STEC counts (AR and NAR) initially increased by 1 to 2 log_10_ CFU g^−1^ during cheese-making. Thereafter, the populations stabilized during salting/drying and then decreased during the early stages of ripening. Exposing the STEC strains in artificially inoculated cheeses to simulated gastric fluid (SGF - pH: 2.0) reduced the number of NAR strains to undetectable levels within 40 minutes, versus 120 minutes for the AR STEC strains. AR and NAR STEC were able to survive during the manufacture and ripening of Camembert cheese prepared from microfiltered milk with no evidence of induced acid tolerance in NAR STEC strains.

## 1. Introduction

Shiga-toxin- (Stx-) producing *Escherichia coli* strains, including *E. coli* O157:H7, are a worldwide cause of human disease with a wide spectrum of symptoms ranging from mild diarrhea to life-threatening hemolytic-uremic syndrome (HUS). The Stx-producing family of human disease-associated *E. coli* is also characterized by its diversity of toxin type (i.e., lysogenic either for the Stx1-encoding phage or the Stx2-encoding phage, or with both lysogens) and by its O:H serotype range [1, 2].

In spite of diverse virulence characteristics, one common trait that emerges very clearly is that most of these strains have the ability to withstand gastric acidity [3–6] or the conditions in acidic foods [7–9]. It is noteworthy that acid tolerance plays a vital role in the survival and virulence of diarrheagenic *E. coli* strains [10–12]. The ability of *E. coli* O157:H7 strains to survive in acidic conditions has been studied extensively [13–16], but there are few reports about the tolerance of non-O157 STEC serogroups to organic acids in foods [17, 18]. It is thought that acid resistance and/or induction of acid tolerance may enable pathogens to survive gastrointestinal acidity better and so, ultimately, cause disease [19–22]. Cattle are considered to be a major reservoir of *E. coli* O157:H7 for human infection [23]. The pathogen has been isolated from raw milk [24], and multiple outbreaks of *E. coli* O157:H7 linked to the ingestion of raw milk and dairy products have been reported [25, 26]. Several authors have studied the ability of *E. coli* O157:H7 to grow and survive in different types of cheese. In fresh cheese, *E. coli* O157:H7 increased by 2 log (CFU per gram) during cheese manufacture [27], but total inactivation was obtained during heat treatment (57°C for more than 1.5 hours) of the curd and whey. Reitsma and Henning (1996) found that this microorganism was able to grow during Cheddar cheese manufacture, even with an initial inoculum in milk of only 1 CFU mL^−1^. *E. coli* O157:H7 also survived the manufacture and storage of Camembert and Feta cheeses at 2 ± 1°C for 65 and 75 days, respectively [28].

Moreover, this pathogen was able to survive all stages of smear-ripened cheese production for up to 70 days postmanufacture [29].

*E. coli* O157:H7 was not eliminated from goat's milk lactic cheese, made with raw milk, because this organism tolerates the low pH [30] and low temperatures [31, 32] which characterize the manufacturing and ripening process.

Surprisingly, studies on contamination of milk, or its products, with non-O157 *E. coli* have been limited. Two studies from France have reported STEC prevalence in cheeses [33, 34], 3 studies reported results for ewes' and caprine milk cheeses [35–37] and, very recently, a study described the prevalence of STEC in Swiss raw milk cheeses [38]. Moreover, there are no published experiments evaluating the growth or survival of non-O157 STEC in cheeses.

However, non-O157 *E. coli* infections are, within European member states, considered to be at least as important as *E. coli* O157:H7 infections though they are thought to be generally underdiagnosed [39]. For example, in Italy [40], Denmark [41], and Germany [42], more than 40% of the confirmed cases of STEC-related HUS were caused by non-O157 STEC. More recently, in 2005, French raw milk Camembert-type cheeses contaminated by *Escherichia coli* O26 and O80 caused 16 HUS cases and a national and international recall of the entire production of cheeses [43]. Other human infections with non-O157 STEC from dairy products are well documented [44–47]. These outbreaks suggest that the acid tolerance of the non-O157 STEC strains, like the O157 STEC strains, enables these bacteria to survive in moderately acidic food. Acid tolerance is defined by the growth of log-phase cultures at a moderately low pH (pH 5.5 to 6.0) inducing mechanisms of survival in the more extreme acid conditions of pH 2.5 [48]. A similar phenomenon, termed the “acid tolerance response,” has been demonstrated in *S. typhimurium* [49, 50].

The purpose of the present work was to address the question of whether the acidic resistance confers an ecological superiority. This potential ecological superiority of STEC strains was evaluated by the ability of acid-resistant (AR) and non-acid-resistant (NAR) STEC strains to survive the fermentation process of Camembert-type cheeses.

In a previous study, we have investigated the fate of *E. coli* O157:H7 during the manufacture and ripening of raw goat's milk lactic cheeses and noted that *E. coli* O157:H7 was able to survive the cheese-making process [51].

The main objectives of the present study were firstly to evaluate the growth and survival of AR and NAR Shiga-toxin-producing *E. coli* strains and, secondly, to check whether microfiltered milk Camembert cheeses could induce acid tolerance in inoculated NAR STEC strains.

## 2. Materials and Methods

### 2.1. Bacterial Strains and Culture Conditions

A collection of 62 STEC strains were isolated during previous French epidemiological studies, whose purpose was to determine STEC contamination prevalence in dairy products, in pork, and in the environment [34, 52–54].

With the aim of evaluating the ability of these bacteria to survive exposure to acid, we used the protocol described by Castanie-Cornet et al. [10], in which three AR mechanisms were tested. These mechanisms are as follows:

- AR1: oxidative system (glucose–repressed system),
- AR2: system depending on the presence of glutamate,
- AR3: system depending on the presence of arginine.

A fourth mechanism is a fermentative acid resistance system. It was tested according to the method previously described by Large et al. [55].

Four AR STEC and four NAR STEC strains were selected from a previous study (data not shown). The survival rate of the strains in the presence of glucose and amino acids is displayed in Table 1. Two spontaneous nalidixic acid-resistant mutants were selected in vitro for each AR and NAR STEC strain, previously selected by plating STEC on BHI agar containing nalidixic acid (0.1 to 10 *μ*g/mL) in increasing concentrations, using the protocol described by Truong et al. [56]. Three rifampicin and three spectinomycin mutants were selected using the same protocol. The antibiotic susceptibility of these AR or NAR mutants is shown in Table 1. To follow the growth of these strains during the manufacture and ripening of the Camembert cheese, spontaneous antibiotic-resistant derivatives (nalidixic acid, rifampicin, and spectinomycin) were isolated. We carefully checked that the chosen resistant derivatives were neither affected in their growth rate nor in their acid-resistance properties (data not shown).

### 2.2. Microfiltered Cow Milk

Raw cow milk samples were collected aseptically from the bulk storage tank after it had been cooled to <5°C and maintained refrigerated at 3°C for transportation to Actilait. Cheeses were made with raw cow milk standardized [57] by microfiltration. Raw milk was microfiltered at 40°C through a 1.4 *μ*m ceramic Membralox membrane (Pall Exekia, Bazet, France).

The microfiltered cow milk was analyzed, in order to check that it was not contaminated by STEC, by performing a Polymerase Chain Reaction (PCR) for Shiga-toxin coding genes (*stx* genes) after an enrichment step, as described by Fremaux et al. [58].

### 2.3. Inoculation Procedure and Preparation of the STEC Mixtures

Cultures of antibiotic-resistant STEC strains were maintained at −80°C in cryopreservative beads (STARLAB, Bagneux, France). Prior to preparing the inoculation mixtures, each culture of antibiotic-resistant STEC strains was incubated overnight (18 hours) in LB broth at 37°C. Strains were grown individually at 37°C for 24 hours in LB broth, with or without antibiotics, to reaffirm that the strains retained resistance. All overnight cultures were centrifuged at 8000 × g for 10 minutes. Cells were washed twice in 0.1% peptone water and suspended in 0.1% peptone water to achieve the required inoculation (10^6^ CFU mL^−1^) level by standardization of the absorbance at A_600 nm_ using a spectrophotometer (BioPhotometer, Eppendorf, Le Pecq, France). Mixtures were prepared by combining the 3 adequate STEC strains in equal amounts. They were designed corresponding with the need to have 3 different antibiotic-resistant or non-resistant phenotypes in the same mixture. The 6 mixtures of STEC strains used for this experimentation are detailed in Table 2.

The STEC population in each mixture was confirmed by plating 100 *μ*L of the suspension onto LB-A plates supplemented with the appropriate antibiotic to verify the initial inoculum levels. Six millilitres of mixture were inoculated into 6.5 L of milk to obtain a final concentration in the microfiltered milk of approximately 10^3^ CFU mL^−1^ of STEC.

Twelve batches of cheeses were prepared (two batches per mixture) and 4 batches of uninoculated cheese samples served as negative controls (1 batch per manufacturing day).

### 2.4. Cheese Manufacture

Artisanal microfiltered cow's milk lactic cheeses were prepared following the industrial specifications of the French Institute of cheese (Actilait).

The milk was inoculated (2 × 10^11^ CFU per 100 kg) with mesophilic starters MM100 (*Lactococcus lactis* subsp. *lactis*, *cremoris*, and *lactis* biovar *diacetylactis*), (Rhodia, Dangé-Saint-Romain, France) as well as ripening flora *Penicillium camemberti* (Pc P9, 4 doses per 1000 kg Cargill, St Germain en Laye, France), *Geotrichum candidum* (GCA, 2 doses per 1000 kg, Cargill) and 10.5 mL of a solution of CaCl_2_ was also added per 100 kg of milk. The inoculated milk was matured for 2 hours at 32°C and renneting was carried out using 30 mL of rennet per 100 L of milk (at 530 mgL^−1^ of chymosin, Berthelot, ABIA S.A. Meursault, France). The milk coagulated after 8 minutes and was left undisturbed until the pH decreased to 6.15. The curd was cut into 3 cm cubes, healed for 30 minutes (pH minimum = 6.00), drained and transferred to 77 × 110 mm cylindrical plastic moulds. Cheeses drained for 20–22 hours at 20°C were turned twice during this time. Cheeses weighing approximately 250 g were removed from the mould, and the temperature of the cheese-making chamber was reduced to 20–22°C. After 20 hours, the cheeses were plunged into a saturated brine solution at 10°C for 25 minutes. Cheeses were dried at 13°C for 5 hours and matured at 11°C and 95% relative humidity for 20 days, then finally packaged and stored at 4°C.

**Figure 1:**
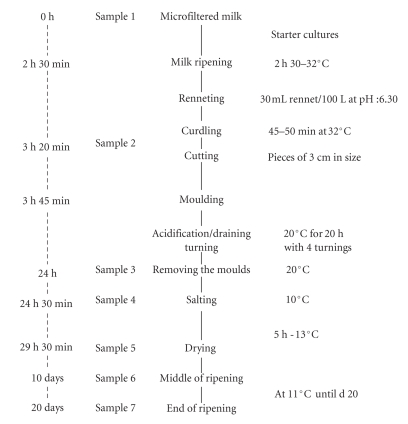
Flowchart for the manufacture of lactic cheeses made with microfiltered milk.

The manufacturing protocol for lactic cheeses made with microfiltered is outlined in Figure 1.

### 2.5. Sampling Steps

Physicochemical and bacteriological analyses were performed at the following steps.

Step 1: Microfiltered milk before the maturation stage.
Step 2: Curd during draining (3 hours 20 minutes).
Step 3: Cheese at the end of the moulding stage (3 hours 45 minutes).
Step 4: Cheese after salting (1 day).
Step 5: Cheese after drying (1 day).
Step 6: Cheese at the middle of the ripening stage (10 days).
Step 7: Cheese at the end of ripening (20 days).

### 2.6. Microbiological Analysis

During cheese manufacture, 25 mL samples of microfiltered milk or 25 g of drained curd or cheese (rind and core) were sampled and homogenized with 225 mL of Buffered Peptone Water (BPW, bioMérieux, Marcy l'Etoile, France) in a sterile bag filter (BagSystem 400 mL Model+, Interscience, Saint Nom la Breteche, France) and stomached for 30 seconds (Stomacher Mix1, AES Laboratory, Bruz, France). The filtered liquid was diluted in BPW and spread, using spiral plating (WASP Spiral plating, AES Laboratory, Bruz, France), onto LB-A plates. LB-A plates were supplemented with rifampicin (100 *μ*g mL^−1^), spectinomycin (100 *μ*g mL^−1^), or nalidixic acid (40 *μ*g mL^−1^), when appropriate, and incubated for 24 hours at 37°C. Enumeration of the colonies was performed with an automatic colony counter EC2 easy count 2 (AES Laboratory, Bruz, France).

### 2.7. Physical-Chemical Measurements

The pH was measured for each cheese (inoculated or not inoculated) at each time of sampling. For the chemical measurements, analyses were performed on the raw milk after microfiltration and on the noninoculated (negative controls) cheeses after 1 day (before brining) and 20 days (end of the ripening stage). Milk fat content was determined by the acido-butyrometric method of Gerber, according to AFNOR (NF V 04-210), and the milk protein rate was obtained using the amido black method (AFNOR, NF V 04-216). Cheese moisture content was determined with an infrared-dryer (Précisa XM60), according to AFNOR (NF V 04-282), and the cheese fat content was measured by the Heiss butyrometric method [59]. The ripened cheese salt content was determined using a chloride analyzer (AFNOR, NF V 04-288).

Cheese pHs were measured using a penetration electrode (pH meter 330, Fisher Bioblock Scientific, F67403 Illkirch Cedex, France).

### 2.8. Exposure of STEC, Present in Artificially Inoculated Cheeses, to Simulated Gastric Fluid (SGF)

Simulated gastric fluid was prepared accorded to the protocol used by Yuk and Marshall [60]. More precisely, simulated gastric fluid consisted of 8.3 g L^−1^ of proteose-peptone (Fluka-Biochemika, Switzerland), 3.5 g L^−1^ of D-glucose (Fluka-Biochemika, Switzerland), 2.05 g L^−1^ of NaCl (Sigma-Aldrich, Lyon, France), 0.6 g L^−1^ of KH2PO4 (Merck Sharp & Dohme, Paris, France), 0.11 g L^−1^ of CaCl_2_ (Sigma-Aldrich, Lyon, France), 0.37 g L^−1^ of KCl (Sigma-Aldrich, Lyon, France), 0.05 g L^−1^ of ox bile (Sigma-Aldrich, Lyon, France), 0.1 g L^−1^ of lysozyme (Sigma-Aldrich, Lyon, France), and 13.3 mg L^−1^ of pepsin (Sigma-Aldrich, Lyon, France). The final pH was adjusted to 1.5 using sterile 5.0 N HCl (Merck Sharp & Dohme, Paris, France). All compounds were autoclaved separately, except for the ox bile, lysozyme, and pepsin which were filter sterilized, followed by aseptic mixing.

However, 90 mL of SGF (pH 1.5), at 37°C, were added aseptically to 10 g of inoculated cheese sampled at the end of the ripening stage. To obtain a final inoculated SGF at pH 2.5, the pH of each sample was lowered to 2.5 using (5.0 N) HCl.

For the enumeration of each STEC strain (Table 1) belonging to the different mixtures used for the inoculation of the milk, homogenate cheese samples were taken at regular time intervals. Viable cell densities were determined by spiral plating appropriate dilutions in TS onto LB-A plates containing rifampicin (100 *μ*g mL^−1^), spectinomycin (100 *μ*g mL^−1^), or nalidixic acid (40 *μ*g mL^−1^). LB-A plates were incubated at 37°C for 24 hours and enumeration of the colonies was performed with an automatic colony counter EC2 easy count 2 (AES Laboratory, Bruz, France).

## 3. Results

### 3.1. PH and Physicochemical Properties During Manufacture and Ripening of the Cheeses

Cheese production was performed in a dairy laboratory in order to provide commercial conditions during production. The chemical composition of the cheese samples produced from the microfiltered milks is shown in Table 3. The fat and protein content in the microfiltered milks were 37.00% and 32.58% w/w, respectively.

The pH of the milk was 6.45 ± 0.02. It declined slightly to 6.03 (SD: ±0.02) during the first 4 hours (draining of curdled milk). Then the pH decreased markedly from 6.03 to 4.65 (SD: ±0.05) at the end of moulding step (24 hours). The pH remained almost stable until the 10th day (4.64 to 4.75) (SD: ±0.02) and increased slightly over the last 10 days of the ripening period to a pH of 5.11 (SD: ±0.03) (Table 4).

### 3.2. Survival of STEC During Lactic Cheese Manufacture

STEC was never isolated from any of the milk samples collected from the bulk storage tank nor from any of the negative control cheeses.

There were no differences between the counts of NAR and AR STEC strains during the manufacture and ripening of the microfiltered milk lactic cheeses.

**Figure 2:**
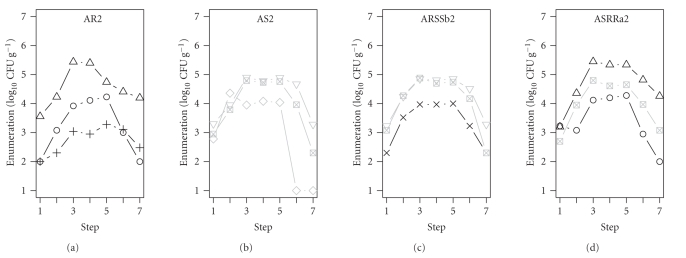
Counts of STEC strains during the cheese manufacture. AR2: 3 AR STEC strains, AS2: 3 NAR STEC strains, ARSSb2: 1 AR and 2 NAR STEC strains, ASRRa2: 2 AR and 1 NAR STEC strains. AR strains: black lines, symbols: ▵, ◯, ×. NAR strains: grey lines, symbols: ▿, ⊠, ⋄. Step 1: milk prior to maturation, Step 3: cheese at moulding stage, Step 5: cheese after drying, Step 7: cheese at the end of ripening (20 days).

As an example, Figure 2 shows the survival of 4 STEC mixtures: the AR2 (3 AR STEC strains), AS2 (3 NAR STEC strains), ARSSb2 (2 NAR and 1 AR STEC strains), and ASRRa2 (2 AR and 1 NAR STEC strains) mixtures during cheese manufacture (Table 2).

In general, whatever the mixture used, STEC counts increased by a range of 1 to 2 2 log CFU g^−1^ during the first steps of the cheese manufacturing and remained relatively stable after salting until the drying stage of the cheeses. Then, during ripening (20 days), the counts of only one NAR STEC strain (346A_Spec_) decreased to 10 CFU g^−1^. The other AR or NAR STEC strains were all counted at levels ranging from 10^2^ to 10^4^ CFU g^−1^ (Figure 2).

### 3.3. Survival of STEC in Simulated Gastric Fluid, pH 2

At the end of ripening (20 days), samples of inoculated cheeses were placed in simulated gastric fluid where the numbers of surviving STEC cells were assessed at 5, 10, 20, 30, 40, 50, 60, 90, and 120 minutes.

Exposure to SGF (pH: 2.5) reduced the number of NAR STEC strains to undetectable levels within 40, 50, and 60 minutes for ANR 42A_Nal_, ANR 418A_Rif_-346A_Spec_, and 360B_Rif_, respectively. In contrast to the NAR STEC strains, all the AR STEC strains survived an exposure of more than 120 minutes in SGF at pH 2.5 (Table 5).

## 4. Discussion

Multiple applications of low levels of acid stress during the life cycles of *Escherichia coli* O157:H7 and other pathogens might increase their likelihood for survival in foods and may enhance the development and establishment of stress-adapted strains with potentially increased virulence in food environments [61–63]. Although acid tolerance in *E. coli* O157:H7 is normally transient, being induced at low pH [14, 30, 64, 65], some outbreak strains (e.g., ATCC 43895) have attained a permanently high, pH-independent acid resistance [17], probably because of an evolutionary response to severe acid stress.

The objective of the present study was to investigate the growth and survival of AR and NAR STEC strains in Camembert-type cheeses. None of our findings have been subjected to a statistical analysis since we have used mixtures of 3 STEC strains for the inoculation of the milk and, hence, some interaction between STEC strains could occur. The use of mixtures, instead of a single strain, is explained by the limited number of batches (16) allowed by Actilait and the decision to study the kinetics of 8 different STEC strains. Moreover, it was not possible to study pathogenic STEC strains, such as *E. coli* O157:H7 or *E. coli* O26, due to the lack of safety level/P3 laboratories.

The number of STEC increased from 1 to 2 log_10_ at the beginning of the cheese manufacture, whilst the pH decreased slightly from 6.45 (milk pH) to 6.03 (first 4 hours). Much of this initial increase could be attributed to the entrapment of STEC in the curd during coagulation followed by further concentration during whey drainage. Then we noted a plateau phase in the growth of STEC from the “cheese at the end of moulding” stage (pH: 4.65) to the “cheese after drying” stage (pH: 4.66). From the middle of ripening (10 days, pH: 4.75) to the end of ripening (20 days, pH 5.11), the STEC population decreased markedly. In much the same way, after 24 hours of manufacture and storage of Camembert cheese inoculated at 10^4^ CFU mL^−1^, Ramsaran et al. [28] observed an increase in *E. coli* O157:H7 counts of about 2 log_10_ . This increase was followed by a decrease in the counts of *E. coli* O157:H7 in all of the cheeses throughout ripening and storage at 2°C.

Vernozy-Rozand et al. [51] showed that significant numbers of viable *E. coli* O157:H7 could be detected in raw goat milk lactic cheeses even 42 days after processing. In Cheddar cheeses, aged for 60 and 120 days, and stored at 7°C, the *E. coli* O157:H7 population was reduced by less than 2 log [66]. Marek et al. [67] reported that *E. coli* O157:H7 could persist in unpasteurized Cheddar cheese whey inoculated at 10^5^ or 10^2^ CFU mL^−1^ for up to 2-3 weeks of storage at 4, 10, or 15°C. The results of Maher et al. [29] showed the presence of *E. coli* O157:H7 even after 90 days in the rind and after 50 days in the core of smear ripened cheese produced from raw milk.

The acid adaptation response is a phenomenon by which microorganisms show an increased resistance to environmental stress after exposure to a moderate acid environment. In this study, the acid adaptation of 4 NAR STEC strains was not induced by the slow and mild acid conditions (4.65–4.75) found during the lactic cheese process because these strains were rapidly destroyed during the strong acid exposure in simulated gastric fluid (pH 2.5).

Hsin-Yi and Chou [68] noted the same effects in fermented milk but a completely opposite effect in acid fruit juice. The authors explained that acid adaptation might lead to an increased susceptibility of the test organism to antimicrobials, such as bacteriocins, hydrogen peroxide, ethanol, and diacetyl, produced by lactic acid bacteria in milk products. The effect of increasing susceptibility to these antimicrobials, due to acid adaptation, may outweigh the effect of enhancing acid tolerance, reducing the survival of acid-adapted *E. coli* O157:H7 in the milk products. Jordan et al. [30] observed that *E. coli* O157:H7 is able to induce an adaptive tolerance response (ATR) when exposed to mild acid conditions, thus conferring a higher resistance on subsequent exposure to strong acid conditions. Bergholz and Whittam [69] studied the survival of enterohaemorrhagic *Escherichia coli* of serotypes O157:H7, O26:H11, and O111:H8 in a simulated gastric environment. Their results indicated that *E. coli* O157:H7 strains were better able to survive in a simulated gastric environment than the STEC strains belonging to the two other serogroups. The authors indicated that this difference was reduced when cultures were held at stationary phase for longer periods of time, suggesting that *E. coli* O157:H7 cells rapidly achieve an enhanced state of AR in the early stationary phase, an ability that may underlie the low infectious dose of this pathogen.

The present study indicates that AR and NAR STEC strains, when initially present at 10^3^ CFU mL^−1^ in milk, would most likely survive artisanal Camembert-type cheese manufacture and ripening (20 days). The biggest decrease was observed for an NAR STEC strain (346A_Spec_) whose counts reached 10 CFU g^−1^ at 20 days. Even if we must keep in mind that the inoculation levels were certainly higher than those observed in naturally contaminated milk, the low infectious dose associated with pathogenic STEC suggests that the 20 day ripening period of these cheeses may not guarantee a safe product for consumers if STEC are present in the raw milk. Consequently, good milk hygiene is crucial in order to reduce the risk of the presence of pathogens in the raw milk cheeses. Moreover, acid adaptation of NAR STEC strains during the manufacture of cheeses prior to their exposure to simulated gastric fluid did not increase the acid resistance of the bacteria. On the basis of these results, additional investigations will be undertaken to evaluate the behavior of STEC during the manufacture of cheeses using a rapid curdling phase linked to a greater drop in acidity than that employed in the present cheese technology.

## Figures and Tables

**Table 1:** Virulence factors and acid resistance of the STEC strains used for the artificial contamination of milk.

	STEC strains			Virulence factors				Acid-resistance				Reference
	Strains	Serotypes^a^	Origins	*eae* *gene* ^c^	*stx*_1_ *gene* ^c^	*stx*_2_ *gene* ^c^	Shiga-toxin producing^b^	AR1	AR2	AR3	AR4	
								rate of survival (%)	rate of survival (%)	rate of survival (%)	rate of survival (%)	

AR STEC strains	ANR	O6:H10	Raw milk	N	P	P	P	96	0,5	0	0	Vernozy-Rozand et al. [34]
	415A_Spec_		cheese									
	ANR	OntH8	Raw milk	N	P	P	P	60	76	0	0	Vernozy-Rozand et al. [34]
	245A1_Rif_		cheese									
	ANR	O166:H28	Environment	N	N	P	P	80	1	0	0	Vernozy-Rozand et al. [54]
	V1_Spec_											
	ANR	O11:H43	Environment	N	P	N	P	67	0	0	0	Vernozy-Rozand et al. [54]
	V10_Nal_											

NAR STEC strains	ANR	O6:H1	Raw milk	N	P	P	P	0	0	0	0	Vernozy-Rozand et al. [34]
	360B_Rif_		cheese									
	ANR	O6:H1	Raw milk	N	P	P	P	0,7	0	0	0,2	Vernozy-Rozand et al. [34]
	42A_Nal_		cheese									
	ANR	O6:H10	Raw milk	N	P	N	P	0	0	0	0	Vernozy-Rozand et al. [34]
	418A_Rif_		cheese									
	ANR	O174:H8	Raw milk	P	P	P	P	0	0	0	0	Vernozy-Rozand et al. [34]
	346A_Spec_		cheese									

^a^Ont: O type not corresponding to any serogroup between O1 and O 174.

^b^P production.

^c^P, positive.

N, negative.

AR1: oxidative system (glucose-repressed system), AR2: system depending on the presence of glutamate, AR3: system depending on the presence of arginine, AR4: system depending on the presence of lysine.

Rif: Rifampicin resistance, Spec: Spectinomicin resistance, Nal: Nalidixic acid resistance.

**Table 2:** Composition of the STEC strains mixtures used for the inoculation of milk.

Cocktail denomination	Cocktail composition	STEC strains
AR1-AR2	3 AR STEC strains	ANR V10_Nal_-ANR 245A1_Rif_-ANR 415A_Spec_
AS1-AS2	3 NAR STEC strains	ANR 42A_Nal_-ANR 360B_Rif_-ANR 346A_Spec_
ARSSa1-ARSSa2	1 AR STEC strains	ANR V10_Nal_
	2 NAR STEC strains	ANR 360B_Rif_-ANR 346A_Spec_
ARSSb1-ARSSb2	1 AR STEC strains	ANR V1_Spec_
	2 NAR STEC strains	ANR 42A_Nal_-ANR 360B_Rif_
ASRRa1-ASRRa2	2 AR STEC strains	ANR 245A1_Rif_-ANR 415A_Spec_
	1 NAR STEC strains	ANR 42A_Nal_
ASRRb1-ASRRb2	2 AR STEC strains	ANR V10_Nal_-ANR 415A_Spec_
	1 NAR STEC strains	ANR 418A_Rif_

**Table 3:** Physicochemical properties of Camembert cheeses.

Component	Cheese at the end of moulding (day 1)	Cheese at the end of ripening (day 20)
Dry matter (% w/w)	38.37 ± 0.49	42.02 ± 0.54
Fat content (% w/w)	19.44 ± 0.31	21.25 ± 0.29
Fat on dry matter (% w/w)	50.65 ± 0.55	50.58 ± 0.40
Moisture content (% w/w)	76.53 ± 0.43	73.65 ± 0.50
Salt content (% w/w)		2.58 ± 0.13
pH	4.66 ± 0.05	5.11 ± 0.03

Average values in 4 uninoculated cheeses as negative controls.

Mean ± standard deviation.

**Table 4:** Changes in pH values during cheese processing.

Stages of sampling							
	Milk before maturation	Curd during draining	Cheese at the end of moulding	Cheese after salting	Cheese after drying	Cheese at the middle of ripening (D+10)	Cheese at the end of ripening (D+20)

Inoculated cheeses	6.45 ± 0.02	6.03 ± 0.02	4.65 ± 0.05	4.64 ± 0.04	4.66 ± 0.05	4.75 ± 0.02	5.11 ± 0.03
Uninoculated cheeses	6.44 ± 0.03	6.02 ± 0.04	4.64 ± 0.06	4.63 ± 0.06	4.65 ± 0.05	4.73 ± 0.04	5.10 ± 0.02

pH values for each cheese, inoculated or not, and at each time of sampling (i.e., 10 pH measures per time of sampling).

Mean ± standard deviation.

**Table 5:** Survival rates of STEC strains in simulated gastric fluid at pH: 2.

Acid-resistance	Strain	Survival rate (%)^a^								
		Time (min)								
		T(5)	T(10)	T(20)	T(30)	T(40)	T(50)	T(60)	T(90)	T(120)

AR	ANR 415A_Spec_	95	95	71	60	50	32	24	10	4
	ANR 245A1_Rif_	86	75	65	63	46	43	36	13	10
	ANR V1_Spec_	97	80	130	115	110	108	105	100	34
	ANR V10_Nal_	81	74	71	66	56	49	29	18	8

NAR	ANR 360B_Rif_	35	13	25	0.37	0.18	0.03	≤0.01	≤0.01	≤0.01
	ANR 42A_Nal_	55	36	6.67	0.31	≤0.01	≤0.01	≤0.01	≤0.01	≤0.01
	ANR 418A_Rif_	66	41	7.18	0.40	0.14	≤0.01	≤0.01	≤0.01	≤0.01
	ANR 346A_Spec_	26	27	59	4	0.59	≤0.01	≤0.01	≤0.01	≤0.01

^a^Percentage of survival calculated as 100× the number of CFU per gram remaining after the acid treatment divided by the initial CFU per gram at time zero.
